# Exploring extracellular vesicle MicroRNAs in Usher syndrome type 1B: Tear-Derived EVs as potential indicators of retinal health

**DOI:** 10.1007/s00018-025-06025-9

**Published:** 2026-01-13

**Authors:** Sander Bervoets, Lonneke Duijkers, Hedwig M. Velde, Zelia Corradi, Edwin M. van Oosten, Nuria Suárez-Herrera, Alejandro Garanto, Miguel A. Moreno-Pelayo, Ronald J.E. Pennings, Rob W.J. Collin, Irene Vázquez-Domínguez

**Affiliations:** 1 Radboudumc Technology Center for Bioinformatics, Department of Medical BioSciences, Radboud University Medical Center, Nijmegen, 6525GA The Netherlands; 2 Department of Human Genetics, Radboud University Medical Center, Nijmegen, 6525GA the Netherlands; 3 Department of Otorhinolaryngology, Radboud University Medical Center, Nijmegen, 6525GA the Netherlands; 4 Department of Pediatrics, Radboud University Medical Center, Nijmegen, 6525GA the Netherlands; 5 Biomedical Network Research Centre on Rare Diseases (CIBERER). Genetics Service, Instituto Ramón y Cajal de Investigación Sanitaria (IRYCIS), Hospital Ramón y Cajal, Madrid, 28034 Spain

**Keywords:** (4–6): Usher syndrome 1B (USH1B), Extracellular vesicles (EVs), Tears, Retinal pigment epithelium, MiRNAs, Biomarkers

## Abstract

Usher syndrome type 1B (USH1B) is a rare inherited disorder characterized by congenital deafness and progressive retinitis pigmentosa, caused by biallelic pathogenic variants in the MYO7A gene. We explored extracellular vesicles (EVs) from two sources: human tears and iPSC-derived RPE cells from USH1B patients and controls. Tear EVs were assessed as a non-invasive biomarker source, while RPE-derived EVs provided insights into disease mechanisms in a controlled, cell-type-specific context. Although RPE differentiation was successful and MYO7A expression levels were similar between patients and controls, Myosin VIIA was not detected by western blot in the patient-derived cells. We examined the EV cargo by small non-coding RNAs (sncRNAs) sequencing from iPSC-RPE apical site and tears to identify molecular signatures of retinal degeneration. Tear EVs showed higher load and diversity of miRNAs than RPE-derived EVs, reflecting a broader ocular origin. Comparative analysis revealed shared retinal sncRNAs (hsa-miR-204, hsa-miR-211, hsa-miR-181a-5p) and group-specific differences. Notably, when comparing to controls, hsa-miR-200a-3p and hsa-miR-194-5p were upregulated in patient tear EVs, while let-7i/c-5p and hsa-miR-320a/b, were downregulated in-patient RPE-derived EVs. Pathway analysis linked these sncRNAs to retinal structure and function, including cytoskeletal remodeling and junctional integrity. Our findings highlight the potential of tear EVs as a non-invasive source of biomarkers that capture retinal molecular alterations in USH1B, with applications for diagnosis, monitoring, and therapeutic development. Although this is a pilot study focused on uncovering promising biomarkers rather than establishing definitive cause–effect mechanisms, it provides a foundation for future research with larger cohorts to validate and expand these findings.

**Graphical Abstract:**

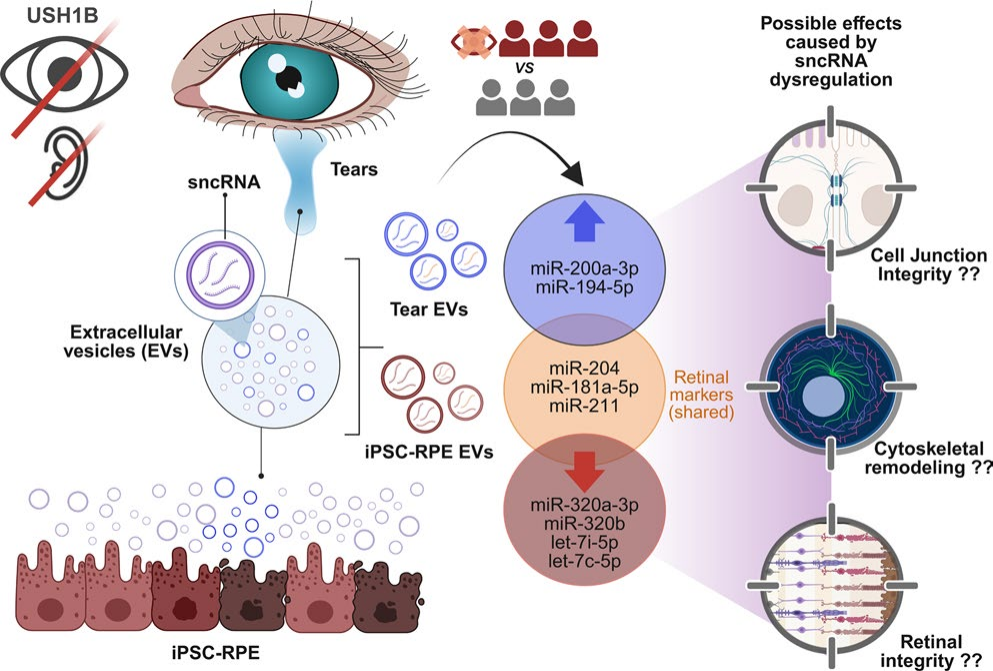

**Supplementary Information:**

The online version contains supplementary material available at 10.1007/s00018-025-06025-9.

## Introduction

Usher syndrome (USH) is the most common form of inherited deaf-blindness, with an estimated prevalence of 1 per 10,000 individuals worldwide [1]. It is a genetically and clinically heterogeneous disorder characterized by the combination of congenital to early-onset sensorineural hearing loss, progressive vision loss due to retinitis pigmentosa (RP), and, in the type 1 form, vestibular dysfunction [2]. Classically, USH is classified into three clinical subtypes: USH1, USH2, and USH3, based on the severity and onset of symptoms [3]. Currently, there are no curative treatments for the RP of USH, and clinical management primarily focuses on supportive therapies such as hearing aids and cochlear implants for hearing loss [4, 5], and low vision aids for vision impairment [6, 7].

Among the subtypes, Usher syndrome type 1b (USH1B) is one of the most severe. Patients with USH1B typically present with profound congenital deafness, vestibular areflexia, and early-onset RP, which generally begins in the first decade of life [8, 9]. USH1B is caused by biallelic pathogenic variants in the *MYO7A* gene [10]. The estimated prevalence of USH1B varies geographically but generally represents around 30–50% of all USH1 cases [11, 12].

The *MYO7A* gene encodes the unconventional myosin VIIA protein, located on chromosome 11q13.5 [13]. Myosin VIIA plays a critical role in the function and organization of stereocilia in inner ear hair cells [14] and is also essential in the retina, particularly in the retinal pigment epithelium (RPE) and photoreceptor cells, where it is involved in opsin transport and phagocytosis of photoreceptor outer segments [15]. Dysfunction of myosin VIIA disrupts these essential processes, contributing to the progressive retinal degeneration observed in USH1B.

Given the absence of effective therapies for retinal degeneration in USH1B, there is a critical need for novel biomarkers to monitor disease progression in the retina, but also therapeutic response. Reliable biomarkers could not only provide insights into the pathophysiology of retinal degeneration but also serve as endpoints in clinical trials for emerging therapies [16].

Extracellular vesicles (EVs), including exosomes and microvesicles, have recently emerged as promising sources of biomarkers across various diseases [17, 18]. Their molecular cargo, which encompasses proteins, lipids, and RNAs, closely reflects the physiological state of their cell of origin [19–21]. Among the different RNA types found within EVs, small non-coding RNAs (sncRNAs) have drawn considerable attention for their roles in regulating gene expression. Within this group, microRNAs (miRNAs) are particularly compelling due to their well-established involvement in posttranscriptional gene regulation and their stability in biofluids, which makes them highly suitable for biomarker discovery [22–24]. Considering their biological relevance, abundance, and technical advantages in terms of detection and quantification, we chose to focus this study specifically on miRNAs as the primary molecular targets. This decision aligns with our aim to identify robust, disease-related biomarkers that are both mechanistically informative and clinically translatable.

In the retina, RPE cells actively produce EVs, which likely contribute to retinal homeostasis and disease. As RP progresses, RPE degeneration may alter EV composition, making them promising biomarker candidates for various conditions [25–28].

EVs are present in almost all biofluids, including blood, urine, saliva, and tears. Tear fluid, in particular, has garnered attention as a non-invasive source of ocular biomarkers. Some studies have suggested that tear-derived EVs may contain retina-related information [29, 30], although conclusive evidence directly linking tear EV cargo to retinal processes is still lacking.

In this pioneering study, we aim to further explore the cargo of EVs isolated from tears of USH1B patients and correlate these findings with EVs derived from human induced pluripotent stem cell (hiPSC)-derived RPE models generated from the same individuals. By including both USH1B patients and genetically unaffected relatives as controls, we seek to evaluate whether tears are a viable and informative source of retina-derived EVs. Ultimately, our goal is to identify suitable novel biomarkers associated with the progression of retinal degeneration in USH1B.

## Materials and methods

### Patient and healthy control selection

For this study, three fully genetically diagnosed USH1B patients from Radboud University Medical Center (Radboudumc) were recruited along with three unaffected relatives, under ethical approval from METC Oost-Nederland (2022–16047). Healthy relatives of the recruited patients were enrolled in the study as controls to ensure the two groups had a genetic context as similar as possible (Table 1). The pathogenicity of the *MYO7A* variants initially designated by clinicians was verified using the online GeneBe prediction tool [31] (GeneBe v0.0.1/4d9a3b4; https://genebe.net) to ensure accurate classification. Recruitment took place from late February to May 2023 to collect blood and tear samples at Radboudumc. All participants were informed about the research goal and the sample collection procedure prior to donating blood and tears. They all agreed to the information and methodology by signing the informed consent forms associated with this project, which adhere to the regulations of the Declaration of Helsinki.

**Table 1:** Genetic characteristics of individuals with Usher 1B syndrome and their unaffected relatives included in this study.

Participant	Sex	Age at collection time	Material(s)	Allele 1			Allele 2			Remarks
				MYO7A variants	Protein domain affected	Pathogenicity prediction	MYO7A variants	Protein domain affected	Pathogenicity prediction	
**Patient 1**	F	20	Tears & blood	c.3719G > A (p.Arg1240Gln)	MyTH4 1	Pathogenic	c.3764del (p.Lys1255Rfs*8)	n.a.	Pathogenic	Compound heterozygous variants.
**Control 1**	F	57	Tears & blood	c.3764del (p.Lys1255Rfs*8)	n.a.	Pathogenic	-	-	-	Heterozygous carrier, mother of patient 1.
**Patient 2**	M	29	Tears & blood	c.4805G > A (p.Arg1602Gln) c.3508G > A (p.Glu1170Lys)	FERM 1 (last aa) MyTH4 1	Benign Pathogenic	c.3719G > A (p.Arg1240Gln)	MyTH4 1	Pathogenic	Compound heterozygous variants
**Control 2**	M	59	Tears & blood	c.3719G > A (p.Arg1240Gln)	MyTH4 1	Pathogenic	-	-	-	Heterozygous carrier, father of patient 2.
**Patient 3**	F	21	Tears & blood	c.5392 C > T (p.Gln1798*)	MyTH4 2	Pathogenic	c.3109–2 A > G (p.?)	n.a.	Pathogenic	Compound heterozygous variants
**Control 3**	F	54	Tears & blood	c.3109–2 A > G (p.?)	n.a.	Pathogenic	-	-	-	Heterozygous carrier, mother of patient 3.
**Patient 4**	M	47	Fibroblasts	c.2461 C > T (p.Gln821*)	n.a.	Pathogenic	c.2461 C > T (p.Gln821*)	n.a.	Pathogenic	Homozygous variants
**Patient 5**	F	39	Fibroblasts	c.5581 C > T (p.Arg1861*)	MyTH4 2	Pathogenic	c.5581 C > T (p.Arg1861*)	n.a.	Pathogenic	
**Control 4**	M	69	Fibroblasts	-	-	-	-	-	-	Obtained from the Stem Cell Facility at Human Genetics dept. (Radboudumc)

Genetic and clinical data of individuals with USH1B and their unaffected relatives. This table summarizes clinical and genetic information from individuals diagnosed with USH1B syndrome caused by pathogenic variants in *MYO7A*, as well as their unaffected relatives. For each participant, sex, age, and the type of biological material collected are indicated. Genetic data include the status of both alleles for *MYO7A* (reference sequence: NM_000260.4), the predicted effect of each variant at the protein level, and their classification according to the GeneBe predictor (https://genebe.net; GeneBe v0.0.1/4d9a3b4), which include the ACMG criteria [31]. USH1B is an autosomal recessive condition that requires two pathogenic variants in *MYO7A* located on different alleles (biallelic) to cause disease. Unaffected relatives are listed as controls and show no clinical signs of retinal disease. Abbreviations: F = female; M = male; fs = frameshift; p.? = unknown effect at protein level; - n.a. = not applicable

The initial sample collection included three patients and three unaffected relatives. However, to enhance the robustness of sncRNA sequencing comparisons, we collaborated with researchers specializing in Usher syndrome at different research centers in Valencia, Spain. These researchers provided fibroblast samples from two additional USH1B patients with *MYO7A* pathogenic variants in homozygosis, being compatible with the study’s objectives (Table 1).

### Tears collection and EV isolation from tears 

On the appointment day, participants donated 4 mL of peripheral blood in heparin tubes, and provided tear samples during the same appointment.

Tears were collected using a novel, non-invasive method involving exposure of the participant’s eyes to continuous airflow to stimulate natural tear production. Tears were then collected directly from the lower lid, which remained open and exposed during airflow application. To minimize irritation, the collection was divided into multiple sessions lasting 2 to 5 min each, with at least 5 min of rest between sessions. After each session, eye drops were applied to hydrate the eyes and prevent any possible discomfort caused by the procedure. A total volume from 400 to 1000 µL of tear fluid was obtained from all participants.

Tear-derived EVs were isolated using a polyethylene glycol (PEG)-based precipitation method developed and optimized at Radboudumc for low-volume biofluids. Adapted from validated PEG workflows for other biofluids [32, 33], this approach was further refined for the limited tear volumes collected, ensuring high EV recovery, preserved vesicle integrity, and optimal RNA yield, where ultracentrifugation is impractical. PEG (Merck Life Science; Darmstadt, Germany) was added to the tear samples at a 1:4 PEG-to-sample ratio, followed by overnight incubation at 4 °C. The samples were then centrifuged at 2000 rpm for 10 min at 4 °C, and the resulting EV pellet was resuspended in 300 µL of filtered 1× PBS. From this suspension, 200 µL were used for RNA isolation and 100 µL were stored at − 80 °C for further analyses.

### Blood collection and PBMC reprogramming to hHiPSC

Peripheral blood mononuclear cells (PBMCs) were isolated at the Stem Cell Technology Center at Radboudumc, within 8 h after blood collection.

PBMCs were reprogrammed into hiPSCs by nucleofection with three reprogramming episomal vectors (pCE-hUL, pCE-hSK, and pCE-hOCT3/4; Addgene, Watertown, MA, USA) using the P3 Primary Cell 4D-Nucleofector X Kit (Lonza; Bassel, Switcherland).

Once hiPSC colonies appeared, they were cultured on growth-factor-reduced Matrigel (Corning, Somerville, MA, USA) in Essential 8 Flex media (Thermo Fisher Scientific; Waltham, MA, USA). Cells were passaged as clumps at a 1:5–1:10 ratio every 5–6 days and maintained at 37 °C and 5% CO_2_. After six passages, the absence of plasmid DNA was confirmed by PCR amplification of the plasmid backbone region. Prior differentiation to RPE, the hiPSC coating was changed to Geltrex (1:100 dilution, Gibco; Grand Island, NY, USA).

### RPE differentiation and EV isolation from RPE

Differentiation of patient and control hiPSCs into RPE cells (hiPSC-RPE) was performed following the protocol published in Regent et al., 2019 [34], with minor modifications.

Briefly, hiPSC-RPE cells were cultured for 110–120 days with media changes every 2–3 days. Cells were passaged three times (P1, P2, P3), during this process the pre-differentiated cells became more pigmented until have a high pigmentation at P3 (higher purity). For passing, hiPSC-RPE cells were first washed with 1x PBS and then incubated twice with TrypLe Express (no phenol red, Gibco) during 10 min at 37 °C to detach those cells not differentiated to RPE. Afterwards, TrypLe Express was removed, a last TrypLe Express cleaning step was conducted, in which RPE cells can be detached and then filtered to remove any presence of the coating. After cleaning steps, cells were counted and seeded at 2 × 10^6^ cells per well concentration (in a 6-well plate). During all the differentiation processes, the media was changed every 2–3 days. At P3, cells were seeded onto transwell membranes (Sterlitech Corporation; Auburn, WA, USA) in a concentration of 1 × 10^6^ cells per 6-well plate transwell membrane to separate apical and basal media compartments.

From three days post-P3 seeding, when the cells were confluent and well-attached, media from the apical and basal sides were collected separately. Collected media were centrifuged at 2000 rpm for 5 min to remove debris, and then stored at −20 °C until at least 80–90 mL of media was accumulated.

### RPE characterization

After completing the RPE differentiation and collecting all the required media for EV isolation, hiPSC-RPE cells were harvested for RNA analysis to assess the expression levels of RPE-specific markers by qPCR. For this, RNA was isolated from both the undifferentiated hiPSCs (day 0) and the differentiated RPE cells (last day of differentiation) using the NucleoSpin RNA kit (Macherey-Nagel, Allentown, PA, USA). One microgram of total RNA was then retrotranscribed using the SuperScript VILO Master Mix (Thermo Fisher Scientific) following the manufacturer’s protocol. Quantitative PCR reactions were performed with the GoTaq Real-Time qPCR Master kit (Promega, Madison, WI, USA) on an Applied Biosystems QuantStudio 5 Digital system. The expression levels of RPE markers were normalized against the housekeeping gene *GUSB*. All primer sequences used are listed in Supplementary Table S1. Expression levels were analyzed using the 2^−(ΔΔCt)^ method, following the approach described by Livak and Schmittgen [35].

In addition to gene expression analysis, RPE morphology and Myosin VIIA localization were assessed by immunocytochemistry (ICC). After media collection, transwell membranes containing the hiPSC-RPE cells were fixed in 4% paraformaldehyde for 10 min at room temperature and washed twice with 1x PBS. The transwell membranes were then cut into 1 cm² pieces and placed onto glass cryoslides with the apical side facing up. To fix the membranes to the glass slides, samples were heated at 60 °C for one hour.

A two-day immunostaining protocol was followed. On the first day, ICC areas were outlined using a hydrophobic pen. Samples were washed three times with 1x PBS and then blocked for 1 h at room temperature in a blocking solution composed of PBS supplemented with 1% Triton X-100, 10% normal goat serum, and 0.3% bovine serum albumin. Primary antibodies, diluted in the same blocking buffer, were added to the samples and incubated overnight at 4 °C. On the second day, the samples were washed five times with 1x PBS. Then, secondary antibodies, also diluted in blocking solution, were applied and incubated for 2 h at room temperature. Afterward, samples were washed with 0.02% Tween-20 in 1x PBS and mounted with cover glasses. Imaging was performed using a Zeiss Axio Imager Z1 Fluorescent microscope (Zeiss, Aalen, Germany). The antibodies used, along with their dilutions and combinations, are listed in Supplementary Table S2.

Finally, the levels of the Myosin VIIA were assessed using western blot analysis. Briefly, hiPSC-RPE cells were washed with 1x PBS and then detached using ReLeSR (StemCell Technologies, Vancouver, Canada). The pellets were washed with 1x PBS prior to freezing using liquid nitrogen. The defrosted pellets were then incubated in a modified radioimmunoprecipitation assay (RIPA) buffer containing 50 mM Tris-HCl (pH 7.5), 150 mM NaCl, 1% NP-40, 0.75% SDS, 0.5% sodium deoxycholate, and 1 mM EDTA, supplemented with a 1x complete protease inhibitor cocktail (Roche, Basel, Switzerland), for 15 to 30 min at 4 °C, followed by homogenization via sonication. Total protein was quantified using a Pierce BCA Protein Assay Kit (Thermo Fisher Scientific), following the manufacturer’s instructions. Then, 20 µg of total protein was loaded onto a Mini-PROTEAN TGX Stain-Free 4–15% gel (Bio-Rad, Hercules, CA, USA), and subsequently transferred onto a nitrocellulose membrane (TransBlot Turbo Transfer Pack, Bio-Rad). The membranes were blocked for 1 h at room temperature with blocking buffer consisting of 5% bovine serum albumin (BSA) (Sigma-Aldrich) in T-PBS (phosphate phosphate-buffered saline plus 0.2% Tween 20) and incubated overnight at 4 °C with primary antibodies (see Supplementary Table S2) diluted 1:1000 in blocking solution. Unbound antibodies were washed three times for five minutes with T-PBS, followed by incubation with secondary antibodies (see Supplementary Table S2) diluted 1:10,000 in blocking solution. The membranes were scanned with an Odyssey CLX (LI-COR Biosciences). Semi-quantification of the observed bands was performed using Fiji 1.53. The β-Tubulin (50 kDa) signal was used to normalize the input amount.

### EV isolation from hiPSC-RPE media

Ultracentrifugation was used for iPSC-RPE media, where the large available volume allows high-purity EV isolation and complements the PEG-based approach used for tear samples. Once the required volume (80–100 mL) of apical and basal media was collected for each cell line, EVs were isolated by ultracentrifugation. First, each apical or basal media was centrifuged separately at 2,000 × g for 20 min at 4 °C to remove cells and large debris. The resulting supernatant was divided equally between two ultracentrifuge tubes to ensure processing of the entire volume collected for each section. The samples were then centrifuged at 12,000 × g for 30 min at 4 °C to eliminate larger vesicles and apoptotic bodies.

Following this step, the supernatants were filtered through a 0.22 μm syringe filter to further remove any remaining contaminants. The filtered media were then ultracentrifuged at 100,000 × g for 70 min at 4 °C to pellet the EVs. After ultracentrifugation, the media were carefully removed, and the EV pellets were resuspended in cold, filtered 1x PBS.

To further purify the EVs, the resuspended samples underwent an additional wash step by centrifuging again at 100,000 × g for 70 min at 4 °C. Finally, the supernatant was discarded, and the EV pellets were resuspended in 100 µL of cold, filtered 1x PBS and stored for subsequent characterization analyses.

### Tears- and hiPSC-RPE EV characterization

EVs were characterized by size using a Nanosight NS3000 (Malvern Panalytical, Malvern, Worcestershire, UK). To ensure the measurements were within the optimal particle detection range, the EVs were diluted five- to tenfold and measured twice [36]. Samples were analyzed according to published recommendations [37, 38].

To assess the presence of tetraspanins (EV surface marker proteins such as CD9, CD63, CD81) on the EV surface, a dot blot assay was performed. Briefly, between 0.5 and 2 µL of each sample was spotted onto a nitrocellulose membrane, ensuring a comparable particle range across all samples. Filtered 1x PBS was used as a negative control. After air-drying, the membrane was blocked with 5% non-fat dry milk in 1x PBS for 1 h at room temperature. The membrane was then washed three times with 0.2% Tween-20 in 1x PBS.

Next, the membrane was placed in a humidified chamber, and primary antibodies (kindly supplied by the María Yañez-Mo laboratory (Madrid, Spain)) were applied directly onto each sample spot. Samples were incubated overnight at 4 °C. On the following day, the membrane was washed three times with 0.2% Tween-20 in 1x PBS, then incubated with the appropriate secondary antibody. Information about the employed antibodies and their conditions can be found in Supplementary Table S2. After another three washes, the blots were developed using the Odyssey Imaging System (Li-Cor Biosciences, Lincoln, NE, USA). The detected signals were semi-quantified using Fiji software [39], and normalized to total particle number per sample. The whisker plots represent normalized fluorescence intensity per 10⁸ particles.

### RNA isolation from the EVs

RNA from EVs was extracted using the miRNeasy Serum/Plasma Kit (QIAGEN, Hilden, Germany) following the manufacturer’s protocol. RNA was resuspended in 30–40 µL of RNase-free water. The presence and quality of small RNAs, including miRNAs, were assessed using the Agilent TapeStation system following the High Sensitivity RNA ScreenTape assay instructions.

### Short Non-Coding RNA (sncRNA) library Preparation and sequencing

EV-derived RNA was used for sncRNA library preparation using the TruSeq Small RNA Library Preparation Kit (Illumina, San Diego, CA, USA), following the manufacturer’s instructions. As the input material consisted of purified RNA, the final clean-up steps recommended in the standard protocol were omitted. Prepared libraries were sequenced on an Illumina NextSeq 2000 platform using the following configuration: Read 1, 59 bp; Index 1, 10 bp; Index 2, 10 bp; Read 2, 59 bp.

### Data analysis

For the primary data processing, data QC, and bioinformatics analysis, we applied the NF-core [40] community standardized Small RNA-Seq pipeline [41] (smrnaseq, version 2.2.4) based on Nextflow DSL2 (version 23.10) [42]. Part of this pipeline are the tools; multiqc (version 1.15), edgeR (version 3.36.0), limma (version 3.50.0), bioconvert (version 0.4.3), mirdeep (version 2.0.1), mirtop (version 0.4.25), seqkit (version 2.3.1), fastqc (version 0.11.4), and samtools (version 1.17). Plots that were generated from the primary data processing are made using the R project (version 4.0.3) and the packages ggplot (version 3.1.1) and pheatmap (version 1.0.1).

Smrnaseq 2.2.4 produced raw expression count table, that we subsequently process as the basis for differential expression analysis using R (version 4.4.1) and differential expression analysis package edgeR (version 4.0.2) combined with the packages ggplot (version 3.1.1) and pheatmap (version 1.0.1). EdgeR applies sample normalization, dispersion compensation, a general linearized model, and a likelihood ratio test to identify differentially sncRNAs. The p-values were corrected for the false discovery rate (FDR) using Benjamini-Hochberg. Lowly expressed sncRNAs (counts per million < 1 for sncRNAs, summed over all samples) were removed from the analysis. For downstream analysis, namely GO term and Pathway Enrichment analysis, we use Logarithmic fold change (LogFC) for differing expression levels. To associate microRNAs to their respective target genes, we used packages miRBaseConverter (version 1.34) and miRNAtap (version 1.43). For the actual GO and Pathway enrichment we used the package clusterProfiler [43] (version 3.21) on the org.Hs.eg.db database.

In addition to the primary bioinformatic analysis described above, we employed the other online platforms to validate the differential expression of sncRNAs between groups and to explore potential interactions relevant to retinal dystrophy.

First, we used the Babelomics platform (http://babelomics.bioinfo.cipf.es/) [44] to support our initial findings. RNA sequencing data, previously processed and compiled into a data matrix, were analyzed by comparing different sample groups (e.g., control vs. patient, and RPE vs. tears). The analysis within Babelomics was performed using the Trimmed Mean of M values (TMM) normalization method [45], and significance was assessed using false discovery rate (FDR) using Benjamini-Hochberg correction for multiple testing. Comparisons with an adjusted p-value of ≤ 0.05 were considered statistically significant. In addition to this, and in order to improve data visualization and the comparison between samples, all heat maps were generated using the BioRender Graph Design tool (https://app.biorender.com/). The number of reads per miRNA was used as input, and the data were normalized by column (miRNAs) to facilitate comparison of expression differences between samples, regardless of the endogenous expression levels of each individual miRNAs. The resulting heat maps provide a color-based representation of relative expression and do not involve any statistical analysis.

Following the identification of differentially expressed miRNAs, we used miRNet (https://www.mirnet.ca/upload/MirUploadView.xhtml) [46] to investigate potential shared gene targets of these miRNAs. Finally, the predicted target genes were analyzed using STRING (https://string-db.org/) [47] to identify functional clusters and pathways that may be regulated by the dysregulated sncRNAs.

## RESULTS

### Patients Show the Expected ***MYO7A***-Associated Phenotype with Identified Pathogenic Variants

All participating patients presented with bilateral sensorineural hearing loss and RP, consistent with the expected phenotype of USH1B. Notably, Patient 3 exhibited more severe visual impairment at the time of sample collection than the other two patients from Radboudumc (patient 1 and patient 2). Comprehensive genetic analysis was performed using Sanger sequencing, which identified the causative pathogenic variants in the *MYO7A* gene in all patients (Table 1). Most of these variants were located within the MyTH4 or MyTH4-FERM domains of the Myosin VIIA. Although these domains are known to mediate interactions with cytoskeletal and membrane-associated proteins, their exact contribution to retinal degeneration remains to be elucidated.

Among all participants, only those recruited at Radboudumc for this study (controls 1 to 3 and patients 1 to 3) provided both blood samples for hiPSC reprogramming and tear samples. In contrast, the external samples (control 4 and patients 4 and 5) yielded only hiPSCs, as tear collection was not possible.

### Consistent RPE Differentiation Across iPSC Lines with Post-Transcriptional Loss of MYO7A in Patient Cells

**Fig. 1:**
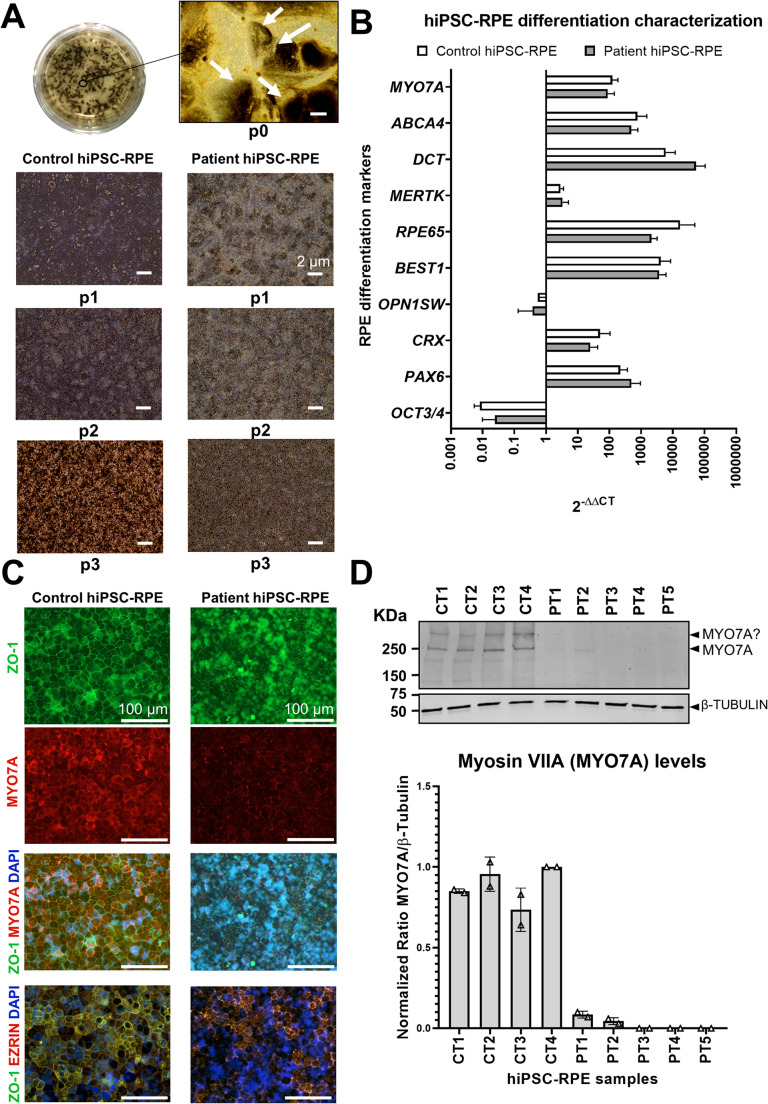
**Characterization of the iPSC-RPE model. (A)** Pigmentation typical of mature RPE cells at passage 0 (P0) is shown, with white arrows indicating the most differentiated areas that were subsequently seeded at passage 1 (P1). Below, representative images depict the evolution of cell morphology and pigmentation in control- and patient-derived RPE lines (scale bar: 200 μm). **(B)** qPCR analysis comparing gene expression levels at day 0 of differentiation and at the time of harvesting (~ day 220). Expression of pluripotency markers (e.g., *OCT3/4*) and photoreceptor-specific markers (e.g., *OPN1SW*) decreased over time, while RPE-specific markers (e.g., *MERTK*, *RPE65*) increased. *MYO7A* expression was also assessed, showing lower levels in patient-derived lines compared to controls. **(C)** Representative immunocytochemistry (ICC) images of control- and patient-derived iPSC-RPE lines showing ZO-1 (green) and *MYO7A* (red) staining to evaluate cell morphology and protein localization, with merged images also including DAPI nuclear staining (blue). Additional merged images include Ezrin staining (red) as a second marker of RPE cell shape (scale bar: 100 μm). **(D)** Representative western blot for Myosin VIIA (~ 250 kDa) in the iPSC-RPE lines (*n* = 2), showing clear detection in control lines, while signal was weak or absent in patient-derived lines. A faint upper band is also observed in control samples and absent in patient lines. Although its specificity suggests a possible relation to Myosin VIIA, this remains uncertain; hence, it is indicated as “MYO7A?” in the figure. The graph below displays Myosin VIIA ratio with respect the loading control β-Tubulin, then expression was normalized to the sample CT4, without variants in *MYO7A* gene. Data are plotted as the average ± SD. PT means Patient and CT means control

The differentiated cells were characterized for morphology, pigmentation, and expression of key RPE markers (Fig. 1). In all cases, the differentiation was consistent in terms than typical polygonal shape of the RPE cells and pigmentation increased along the passages (Fig. 1A). Similarly, the assessment of hiPSC-RPE P3 differentiation relative to day 0 revealed comparable differentiation levels across all hiPSC-RPE lines. No major differences were observed between control and patient lines, and the differentiation efficiency was consistent with previous experiments. Notably, the expression levels of *MYO7A* remained unchanged in the patient lines, despite the presence of pathogenic variants in this gene (Fig. 1B).

To cells, and assess how the pathogenic variants affect Myosin VIIA location and expression, we analyzed the distribution of RPE marker Ezrin together with the tight-junction protein ZO-1, and the co-localization/expression of Myosin VIIA with Ezrin (Fig. 1C, Supplementary Figure S1). Overall, hiPSC-RPE monolayers from controls and patients showed comparable morphology and junctional organization. While Myosin VIIA signal intensity was lower in patient-derived cells, its subcellular localization (distributed between the membrane and cytoplasm) was similar across groups. As shown in Supplementary Figure S1, ZO-1 and Ezrin signals overlapped within each sample, with the highest intensities at corresponding membrane regions and no consistent differences between controls and patients. Minor background variations likely result from imaging through the transwell membrane, which can retain faint non-specific fluorescence despite thorough washing.

To assess myosin VIIA expression at the protein level, we performed western blot analysis using an anti-MYO7A antibody. This revealed two bands: a prominent band at the expected molecular weight of ~ 220–250 kDa and a fainter upper band, which has previously been reported in mammalian samples by other authors using the same antibody [48–50]. In iPSC-derived models carrying pathogenic *MYO7A* gene variants, both bands were markedly reduced or undetectable, indicating a significant loss of Myosin VIIA (Fig. 1D). The reduction of the upper, weaker band further suggests that it may also be related to myosin VIIA. Notably, qPCR analysis showed no significant differences in *MYO7A* mRNA levels between mutant and control lines (Fig. 1B), suggesting that the observed reduction in protein levels may result from post-transcriptional mechanisms.

### EVs from hiPSC-RPE cells are Preferentially Released Apically and present Comparable Tetraspanin expression to Tear-derived EVs

**Fig. 2:**
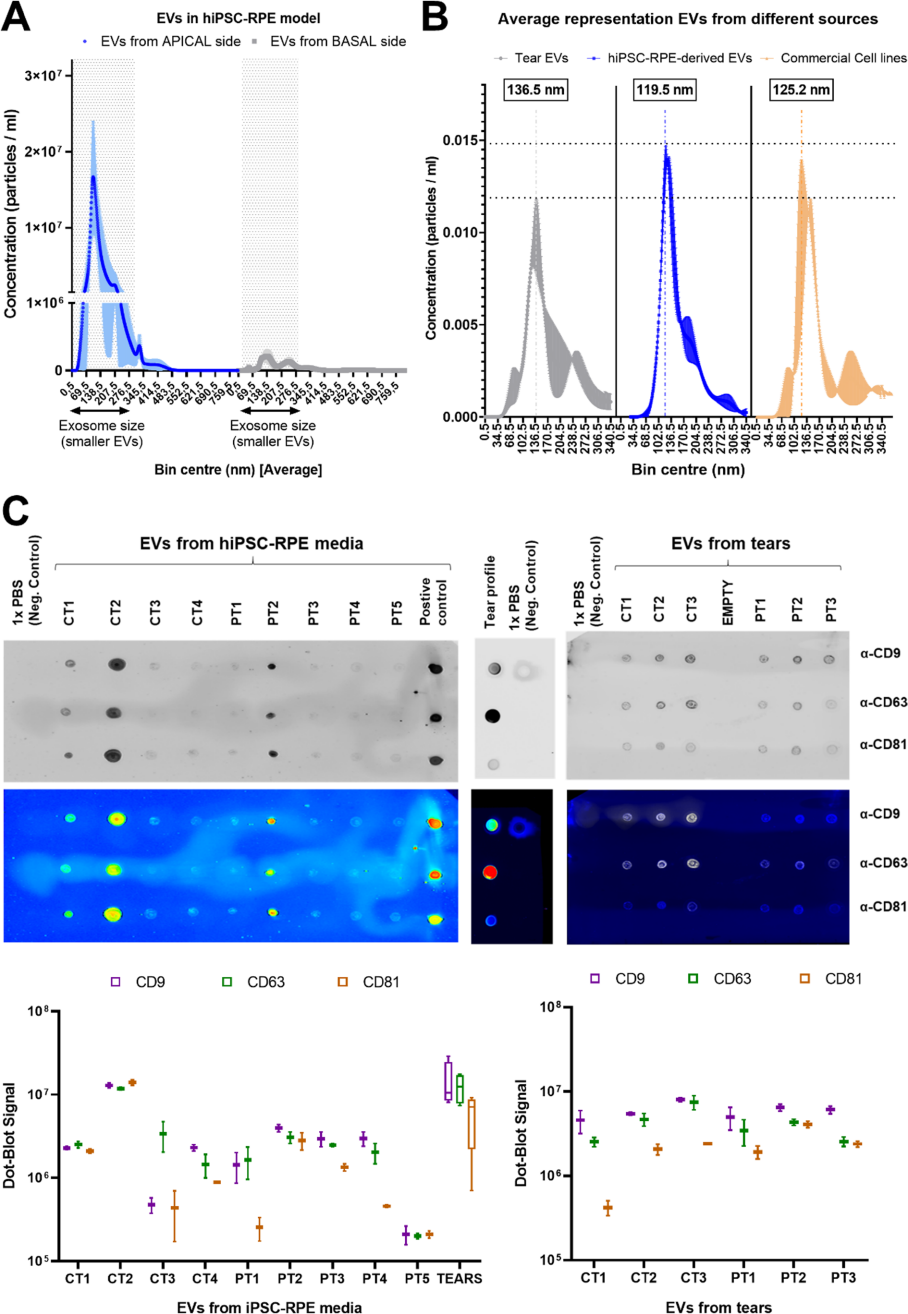
**Characterization of EV properties. (A)** Average size distribution and concentration of EVs collected from the apical (blue) and basal (grey) compartments of the iPSC-RPE model. EVs released from the basal side were produced at very low levels and were therefore excluded from downstream RNA analysis. **(B)** Comparative size profiles of EVs isolated from human tears (grey), the apical media of iPSC-RPE cultures (blue), and commercial cell lines (HEK-293T and ARPE19, in orange). While all sources showed consistent EV patterns, tear-derived EVs exhibited the largest average size, whereas RPE-derived EVs were the smallest. **(C)** Dot-blot analysis of tetraspanin markers (CD9, CD63, and CD81) in EVs isolated from iPSC-RPE media and tears. Due to sample collection differences and size constraints, tear EVs and RPE-derived EVs were analyzed on separate membranes. Filtered 1× PBS was included as a negative control in all experiments. Below, box-and-whisker plots represent normalized fluorescence intensity per 10⁸ particles for each marker across replicates (*n* = 2), showing that EVs from both sources were consistently enriched in CD9 and CD63 compared to CD81. PT means Patient and CT means control

To isolate EVs, iPSC-derived RPE cells were cultured on transwell inserts, which allowed full polarization of the cells, and therefore separate the collected fraction from the apical and basal media. Analysis of the collected samples showed that basal compartments from three independent RPE lines (Patient 1, Control 3, and Patient 3) contained no detectable EVs, while apical compartments consistently contained abundant EV populations. This indicates that EVs are preferentially released from the apical side under our culture conditions **(**Fig. 2A**).** Consequently, all downstream analyses focused on apical EVs.

Particle size analysis revealed no significant differences between EVs from RPE models, in-house commercial cell lines (HEK-293T and ARPE-19, which media was used for methodological control), and tears **(**Fig. 2B**)**, nor between EVs from control and patient-derived RPE cells. Dot blot analysis confirmed the presence of tetraspanins, particularly CD9 and CD63, in all apical EV samples from the RPE models **(**Fig. 2C, **left).** Similarly, tear-derived EVs were also enriched in CD9 and CD63 **(**Fig. 2C, **right).**

### Tear-Derived EVs exhibit higher SncRNA load and diversity than RPE-Derived EVs

**Fig. 3:**
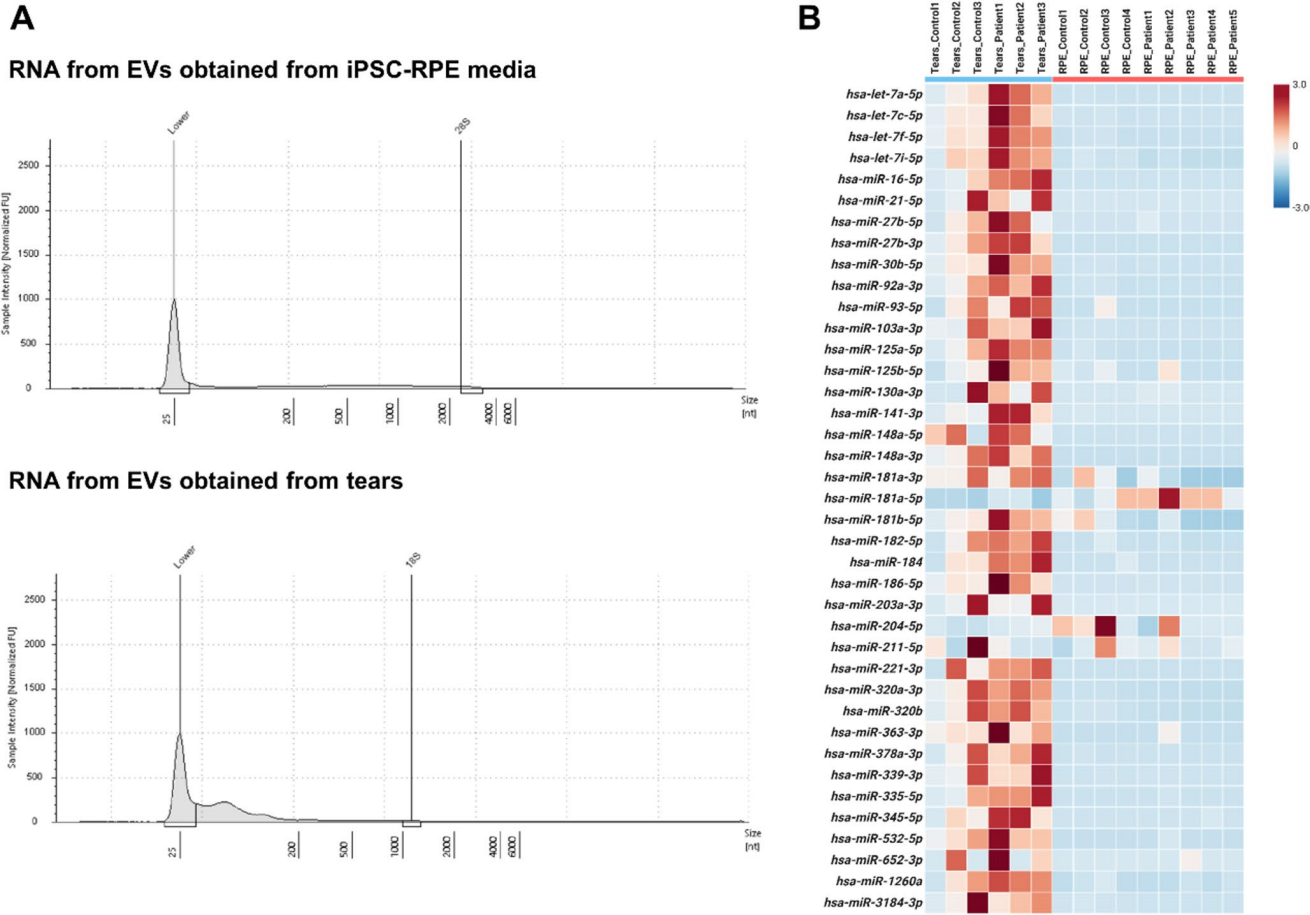
**Characterization of RNA isolated from EVs in this study. (A)** Representative electropherogram profiles obtained by TapeStation analysis of EVs isolated from iPSC-RPE media (top) and human tears (bottom). The migration signal between 24 to 200 is directly related to the amount of sncRNA present in the sample. Based on that, the results suggest that tear-derived EVs are richer in sncRNAs compared to those from the iPSC-RPE model. **(B)** Heatmap showing the distribution and relative abundance of sncRNAs across the different EV samples analyzed, confirming both a higher quantity and greater diversity of sncRNAs in tear-derived EVs compared to those from iPSC-RPE cultures. The heatmap was generated using statistical tools available through the Babelomics platform (http://babelomics.bioinfo.cipf.es/).

Preliminary analyses showed that EVs isolated from tear fluid contained both a higher total sncRNA load and a greater diversity of sncRNA species compared to RPE-derived EVs. This was consistent with TapeStation profiles, where RNA from tear-derived EVs displayed a stronger signal in the region between the lower marker peak at ~ 25 nt and 200 nt, which corresponds to the expected size range of sncRNAs, than RNA from RPE-derived EVs (Fig. 3A). This observation likely reflects the contribution of multiple ocular tissues to the tear EV pool, whereas RPE media-derived EVs represent a more tissue-specific source. Notably, the sncRNA-seq analysis in these samples revealed that the cargo of tear derived EVs were enriched in miRNAs diversity and number of reads (Fig. 3B).

Finally, some miRNAs highly associated to retina or even to specific expression in RPE tissue (Supplementary Table S3) were found not only in the RPE-derived EVs but also in the tears-derived EVs (Supplementary Table S4). However, given the limited number of tear samples (*n* = 3 per cohort), these findings should be interpreted cautiously, as the statistical power was insufficient for conclusive comparisons.

### Distinct SncRNA signatures differentiate control and patient EVs from tears and RPE cells

**Fig. 4:**
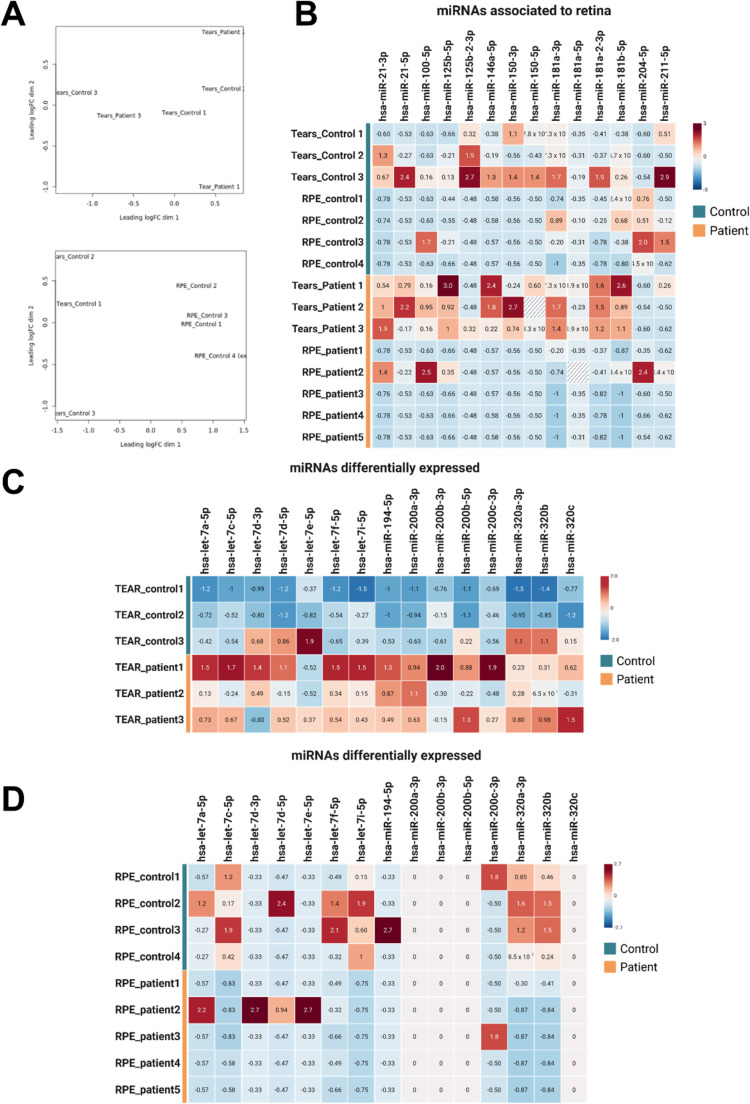
**Comparative analysis of sncRNAs from EVs derived from tears and iPSC-RPE cultures. (A)** Principal component analysis (PCA) plots showing the distribution of sncRNA expression profiles. The upper plot includes all tear-derived EV samples from controls and patients, while the lower plot shows a subset including matched tear and hiPSC-RPE samples from the same individuals (controls 1–3), and control 4 (only RPE sample available). Although tear samples display limited variability, a clear distinction is observed between tear- and RPE-derived EVs. **(B)** Heatmap displaying normalized expression values of selected miRNAs previously associated with retinal tissue across all EV samples. **(C–D)** Heatmaps showing the normalized expression of differentially expressed miRNAs between control and patient samples in EVs from **(C)** tears and **(D)** iPSC-RPE cultures. In all heatmaps, higher expression levels are shown in red and lower levels in blue. Color coding (orange for patients, blue for controls) indicates the clinical group of origin. Heatmaps were created using BioRender.com

We first confirmed that tear samples did not exhibit substantial variability between individuals within the same group (controls or patients), indicating sample consistency (Fig. 4A, **top**). Subsequent analysis of differentially expressed sncRNAs between patients and controls revealed consistent expression changes in both tear- and RPE-derived EV samples. Notably, when comparing the sncRNA expression profiles from tear- and RPE-derived EVs of the same individuals (controls 1–3 and patients 1–3), control and patient samples clustered separately, highlighting clear group-specific expression patterns (Fig. 4A, **bottom**). Next, as tear-EV miRNA enrichment was higher than the one observed in from the EVs of hiPSC-RPE model, we focused on the EV cargo first, and we attempted to find the presence of miRNAs related to the retinal tissue according to the literature (Supplementary table S3, Fig. 4B). Amongst them, we identified in all samples hsa-miR-204, hsa-miR-181a-5p and hsa-miR-211, whose relevance in RPE differentiation and maintenance have been already proved [51–60]. Then, we tried to identify specific differences between patient and control groups. In tear derived EVs from patients, we observed a significant upregulation of hsa-miR-200a-3p and hsa-miR-194-5p (Fig. 4**C** and Supplementary Table S3and S4). In contrast, EVs derived from patient RPE cells showed a significant downregulation of hsa-let-7i-5p, hsa-let-7c-5p, hsa-miR-320a-3p, a microRNA enriched in the retina, and hsa-miR-320b (Fig. 4D; and Supplementary Tables S3 and S4).

### Differentially Expressed sncRNAs either in Tears or hiPSC-RPE Point to Pathways and Molecular Targets Relevant to Retinal Disease

**Fig. 5:**
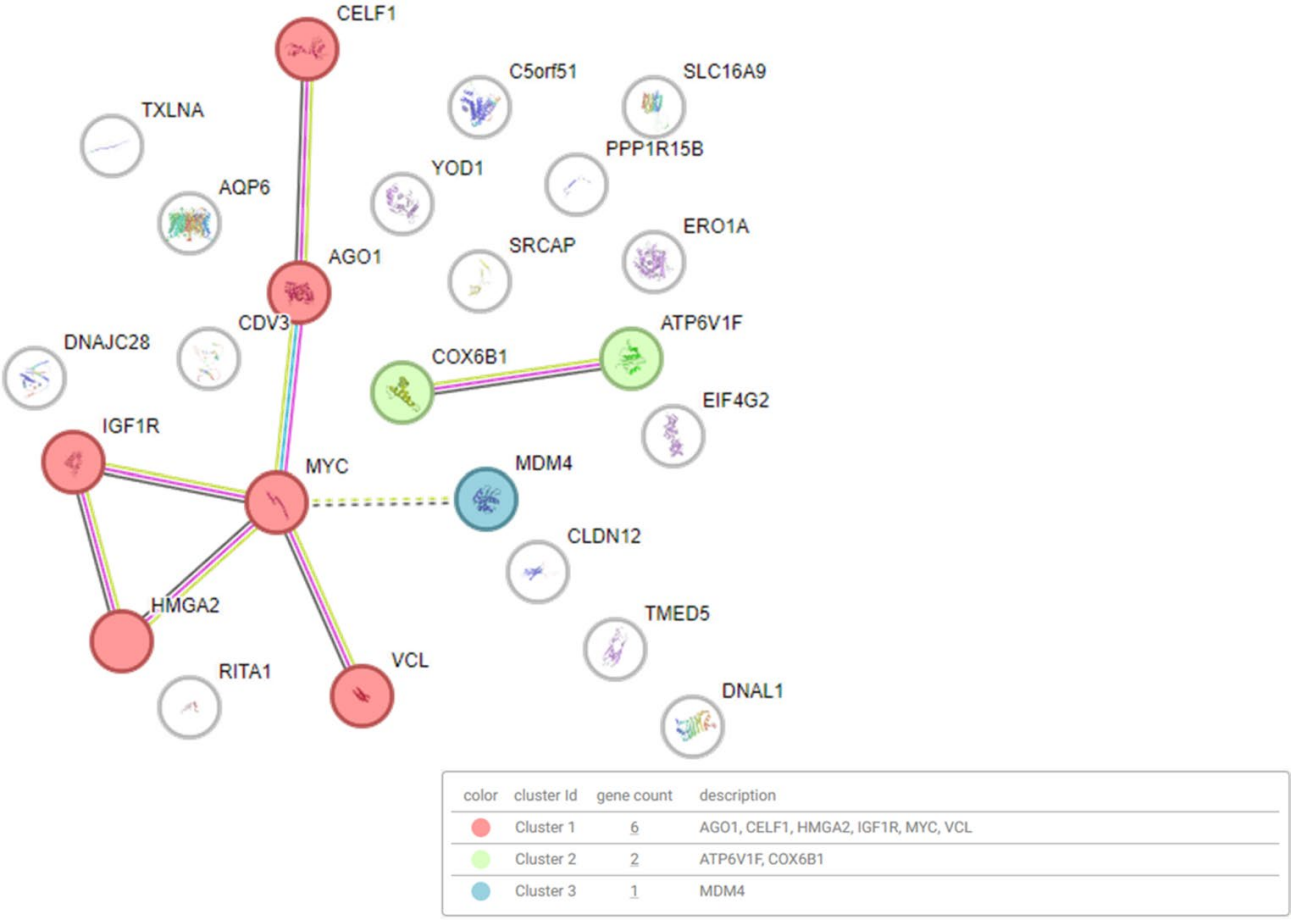
**STRING network analysis of predicted target genes based on miRNet results (Supplementary Figure ****S2****).** The analysis revealed three main clusters of interacting proteins whose expression is potentially regulated by the differentially expressed miRNAs identified in this study. These clusters are highlighted in red (AGO1, CELF1, HMGA2, IGF1R, MYC, VCL), green (ATP6V1F, COX6B1), and blue (MDM4), indicating varying levels of network density and interconnectivity. The red cluster comprises the most interconnected targets, suggesting key roles in RNA regulation, oncogenic signaling, and cytoskeletal organization. Notably, several targets, such as MYC, IGF1R, and HMGA2, are involved in cell proliferation, differentiation, and retinal development, making them particularly relevant for understanding disease-related regulatory pathways in the retina

To understand the potential biological implications of these changes, we conducted pathway enrichment and target prediction analyses using multiple publicly available tools like clusterProfiler [43], miRNAtap [61], and miRNet [46] (Supplementary Figure S2). The sncRNAs differentially expressed in patient samples in comparison to controls were found to be involved in several biological pathways relevant to retinal structure and function, including adherents junctions, proteolysis, and multiple cell signaling cascades (Supplementary Table S5). Remarkably, predicted target genes included AGO1, VCL (which encodes vinculin, which is a known interactor of retinal focal adhesion partners and retinal morphogenesis [62]), and MDM4, a regulator of p53 signaling [63] (Fig. 5). These findings identified molecular candidates potentially modulated by sncRNAs in inherited retinal disease and open avenues for further functional validation.

## DISCUSSION

As a pilot study, this work aims to uncover promising candidate biomarkers rather than establish definitive cause–effect relationships, which would require larger patient cohorts. Nevertheless, it provides novel insights into the potential of tear fluid-derived EVs as a non-invasive source of biomarkers for retinal degeneration in USH1B. By comparing sncRNA profiles, particularly miRNAs, in EVs from both control and patient-derived iPSC-RPE cells and tear samples, we identified distinct molecular signatures that may reflect USH1B pathology. These findings set the stage for future studies with larger cohorts to validate and expand on these observations.

All pathogenic variants identified in our USH1B patient cohort affect the MyTH4 domain of the *MYO7A* gene (Table 1), a region absent in conventional myosins but essential for protein–protein interactions with USH1 complex components such as USH1C [64–66]. These interactions are crucial for the assembly and stabilization of the USH1 complex (Myosin VIIA, USH1C, SANS, CDH23), which maintains the architecture and function of sensory cells in the retina and cochlea [67]. Disruption of this domain may impair intracellular transport and cargo binding [68], processes necessary for photoreceptor renewal and RPE homeostasis [69–71], as well as for stereocilia structure in auditory hair cells [72]. Notably, the most severely affected patient in our Radboudumc cohort, patient 3, carried a pathogenic variant in the MyTH4-2 domain, whereas patients 1 and 2 had variants in MyTH4-1. Although this observation suggest potential subdomain-specific differences, previous studies have not established a consistent genotype–phenotype correlations between MyTH4 subdomains and disease severity in USH1B [73, 74]. Therefore, the more pronounced phenotype of patient 3 may instead reflect individual genetic background or modifier effects, together with the presence of a truncating variant that likely reduces overall Myosin VIIA stability.

Interestingly, MYO7A protein levels appeared comparable among control lines, including those heterozygous for truncating variants. This agrees with previous reports showing that heterozygous carriers often maintain near-normal MYO7A protein levels through compensatory expression or rapid degradation of unstable truncated peptides [75]. Such compensation is consistent with the absence of retinal symptoms in carriers and supports that one functional MYO7A allele is generally sufficient to sustain RPE homeostasis.

Overall, the discrepancy between normal *MYO7A* transcript levels and reduced protein expression in patient-derived RPE cells suggests that post-transcriptional mechanisms contribute to impaired Myosin VIIA synthesis. Possible explanations include nonsense-mediated mRNA decay in transcripts carrying premature stop codons [76] and increased proteasomal degradation of unstable or misfolded proteins [77]. Further studies assessing transcript stability and protein turnover will be essential to clarify these mechanisms and to explore whether specific mutations differentially affect MYO7A expression and retinal function.

In addition to post-transcriptional regulation, our western blot studies consistently revealed two bands instead of the anticipated single band at 220–250 kDa for Myosin VIIA, with only the lower band matching the expected size. The nature of the faint, upper band remains unclear. Although similar observations have been reported by other groups using the same antibody [48–50], its identity has not been definitively established. Although the band migrates close to the expected molecular weight of canonical myosin VIIA and is therefore unlikely to correspond to a dimer, this possibility cannot be entirely excluded given the absence of higher molecular weight markers in our western blot. Some studies suggest that myosin proteins may undergo post-translational modifications that alter migration in SDS-PAGE [78, 79], though no specific modifications have yet been described for Myosin VIIA. Further characterization, will be necessary to clarify its origin and potential relevance.

Setting these brief points aside, we now turn our attention to the EV assessment. Our findings revealed that EVs isolated from tear fluid exhibited a higher total sncRNA load and greater diversity than those from hiPSC-RPE cultures. This likely reflects the broader origin of tear EVs, which includes contributions from both anterior and posterior ocular tissues. An additional factor to consider is that the RPE cells used here were hiPSC-derived, and published studies indicate that their degree of resemblance to adult RPE remains debated, with reports supporting either a more fetal-like state or substantial similarities to adult RPE [80–83]. Therefore, full comparability with adult RPE cannot be assumed, which further underscores the relevance of our findings and highlights the need for future validation using primary RPE sources. Despite this complexity, tear EVs displayed similar features (e.g., size and tetraspanin expression) to those from hiPSC-RPE media (Fig. 2C), suggesting a possible shared source.

Two EV isolation approaches were employed due to the significant differences in the properties of the samples. Tear fluid is only available in small volumes (400–1,000 µL) and contains high proportions of EVs, which makes ultracentrifugation impractical. PEG precipitation enables the efficient recovery of EVs from limited material while preserving their integrity and RNA cargo. In contrast, the iPSC-RPE culture medium was processed by ultracentrifugation, which provides a higher level of purity when larger volumes of EVs are required for further applications. Despite these different approaches, both the tear- and the RPE-derived EVs exhibited comparable particle sizes and tetraspanin profiles (CD9 and CD63; see Fig. 2C), which suggests that both isolation methods are consistent.

One factor that could potentially influence our results is the tear collection procedure. In this study, tear secretion was gently stimulated by brief exposure to continuous airflow to promote natural tear production. Unlike chemical or irritative stimuli (e.g. onion vapors or hyperosmotic eye drops), airflow represents a mild and transient mechanical stimulus that minimizes ocular stress. Each session lasted only a few minutes and was followed by rest periods involving the application of sterile lubricating eye drops to prevent dryness. This solution does not induce tearing, but rather hydrates and cleans the surface of the eye. While this approach is likely to have negligible effects on EV composition, any stimulation may affect tear content. Future studies should therefore compare basal and airflow-stimulated samples in order to standardize collection protocols for biomarker research.

Regarding EV cargo, tear EVs from USH1B patients exhibited a sncRNA profile, specifically of miRNAs, that partially mirrored those of RPE-derived EVs. This suggests that tear-derived EVs may reflect the molecular environment of the entire eye, including the anterior and posterior regions, not just the retina. Therefore, tear fluid may reflect retinal molecular changes. However, larger patient and control cohorts are needed for validation.

The identification of RPE-specific miRNAs such as hsa-miR-204, hsa-miR-211, and hsa-miR-181a-5p [51–60] within EVs isolated from tear fluid in both in patients and controls highlights the potential of tears as a valuable, non-invasive source for biomarker discovery in Usher syndrome. Hsa-miR-204 and hsa-miR-211 are highly enriched in the RPE and are crucial for maintaining its differentiation and function, including photoreceptor survival and visual cycle regulation [56, 84]. Hsa-miR-181a-5p has also been associated with neuroprotective and anti-inflammatory pathways in retinal cells [59]. The detection of these RPE-related miRNAs in tear-derived EVs suggests that molecular alterations occurring deep within the retina can be reflected in a minimally invasive and easily collectable biofluid. This finding not only reinforces the utility of tear EVs for early diagnosis and stratification in retinal dystrophies, but also points to their potential for longitudinal monitoring of disease progression and response to therapy. Furthermore, it opens the possibility of using tear EVs for therapeutic delivery or as targets in miRNA-based interventions, advancing the prospects for personalized treatment in Usher syndrome and related retinal diseases.

Differential expression analyses identified specific miRNAs altered in USH1B. In tear EVs, hsa-miR-200a-3p and hsa-miR-194-5p were upregulated. Hsa-miR-200a-3p has been implicated in retinal diseases such as diabetic retinopathy, where it modulates the TGF-β/Smad pathway, affecting angiogenesis and inflammation [85]. Hsa-miR-194-5p is typically associated with epithelial protection, suppressing epithelial-to-mesenchymal transition (EMT) and regulating ZEB1 and SOX17/VEGF signaling pathways [86, 87]. Its upregulation may reflect a compensatory mechanism in response to RPE dysfunction. Alternatively, as MYO7A is involved in intracellular transport [71], its deficiency could disrupt miRNA loading into EVs, resulting in selective enrichment of hsa-miR-194-5p. Elevated hsa-miR-194-5p may also point to early inflammatory or remodeling processes in the degenerating retina [88]. In RPE-derived EVs, we observed downregulation of hsa-let-7i-5p, hsa-let-7c-5p, hsa-miR-320a-3p, and hsa-miR-320b. These miRNAs are known to influence cell differentiation, proliferation, and retinal vascular function [89–94].

To investigate the biological relevance of miRNA dysregulation in USH1B samples, we performed pathway enrichment and target prediction analyses using the tool miRNet (Supplementary Figure S1). Importantly, both the patient and control cohorts displayed a similar retinal miRNA molecular signature, with a high degree of homogeneity among samples within each cohort for the differentially expressed miRNA. The differentially expressed miRNAs in the patient cohort were enriched in pathways fundamental to retinal tissue integrity, including adherens junction formation, proteolysis, and cytoskeletal signaling, processes that are essential for the maintenance of RPE and photoreceptors (Supplementary Table S5). Notably, several predicted targets, including AGO1, MDM4, and VCL (vinculin), point to mechanisms through which miRNAs may modulate retinal homeostasis (Fig. 5). AGO1, a core component of the RNA-induced silencing complex [95, 96], supports the expected post-transcriptional regulatory role of miRNAs. MDM4, a regulator of p53 signaling [63], is particularly relevant given its developmental expression in the retina and involvement in cell survival pathways. VCL, which encodes vinculin, plays a key role in focal adhesion dynamics by linking talin, β1-integrin, paxillin, and F-actin, thereby stabilizing cell–matrix adhesions and supporting cytoskeletal organization, which are functions critical for retinal morphogenesis and structural maintenance [62]. Together, the convergence of these targets underscores the potential contribution of miRNA dysregulation to USH1B pathogenesis and highlights the need for further functional validation in vitro and in vivo.

Recently, Getachew et al. analyzed sncRNA content of EVs from CRISPR/Cas9-edited *PRPF31+/−* hiPSC-RPE cells to identify biomarkers of autosomal dominant RP [97, 98]. In contrast, we analyzed EV miRNAs from Myosin VIIA-mutant patient-derived iPSC-RPE cells and matched tear samples, using unaffected relatives as controls. None of our differentially expressed miRNAs overlapped with those reported by Getachew et al.; however, comparison with their “enriched” and “depleted” datasets revealed some similarities (Supplementary Table S6). For example, hsa-miR-375-3p was detected in both RPE- and tear-derived EVs, and hsa-miR-378a-3p was low in RPE EVs but higher in tear EVs from USH1B patients. Other miRNAs, such as hsa-miR-4448 and hsa-miR-24-3p, were minimally expressed in our samples, consistent with patterns in their control model (Supplementary Table S4).

Among miRNAs “depleted” in *PRPF31*+/− EVs, we detected hsa-miR-151a-5p and hsa-miR-92a in our samples. MiR-151a-5p was low in tears but absent in RPE EVs, whereas hsa-miR-92a was present in both sources, higher in tear EVs, and lower in RPE EVs, aligning with Getachew et al. [99]. Differences likely reflect disease-specific mechanisms (*PRPF31* vs. *MYO7A*), model systems (isogenic vs. patient-derived), and EV sources (cultured vs. in vivo). Residual presence of miRNAs classified as “depleted” in tear EVs may indicate milder dysfunction or distinct in vivo regulatory dynamics. Overall, our findings emphasize that miRNA composition is strongly influenced by disease type and EV source, with tear-derived EVs potentially capturing subtle in vivo-specific changes (Figs. 3 and 4, Supplementary Tables S4 and S6).

Taken all our and their data together, our results suggest that a subset of miRNAs, such as hsa-miR-92a, hsa-let-7 family members, and hsa-miR-200a-3p, could converge on pathways related to epithelial integrity, inflammation, and vascular homeostasis in retinal degeneration. Their dysregulation in tear- and RPE-derived EVs positions them as candidate biomarkers or therapeutic targets in inherited retinal diseases.

The potential of dysregulated miRNAs as biomarkers is illustrated by studies in other diseases. For example, hsa-miR-122, a liver-specific miRNA, was identified as a circulating biomarker in HCV patients, correlating with liver injury markers and histologic activity [100–102] (https://clinicaltrials.gov/search?intr=Miravirsen). Similarly, hsa-miR-29 is consistently downregulated in fibrotic conditions and regulates collagen production in hepatic stellate cells [103–105]. These findings highlight how miRNA profiling can inform disease mechanisms and identify candidate targets, though therapeutic applications remain exploratory and require extensive validation [106, 107].

In our study, the identification of a disease-associated miRNA signature may not only provide insight into the molecular underpinnings of USH1B but also offer a basis for future therapeutic interventions. Moreover, our data demonstrate the feasibility of using tear fluid-derived EVs to capture retina-relevant molecular signatures that mirror retinal alterations through a non-invasive approach, which is especially suitable for longitudinal patient monitoring. While these findings are promising, the relatively small size of our cohort warrants cautious interpretation. Future studies involving larger and more diverse patient populations will be essential to validate the diagnostic and prognostic potential of these miRNAs, and to further investigate their mechanistic roles in USH1B pathogenesis.

In conclusion, this study highlights the promise of tear EVs as a window into retinal health. The identified sncRNA signatures may enhance our understanding of USH1B pathophysiology and contribute to the development of non-invasive diagnostic tools for retinal degeneration.

## Supplementary Information

Below is the link to the electronic supplementary material.

Supplementary Material 1 (54.8 KB)

Supplementary Material 2 (159 KB)

Supplementary Material 3 (453 KB)

## Data Availability

sncRNA transcriptome data is available at EGA (id data: EGAS50000001200). Other data are available from the corresponding author upon reasonable request.
