# Supplementary material for: Exploring extracellular vesicle MicroRNAs in Usher syndrome type 1B: Tear-Derived EVs as potential indicators of retinal health
Source: Cell Mol Life Sci. 2026 Jan 13;83(1):75. doi: 10.1007/s00018-025-06025-9 (PMC12858694; doi:10.1007/s00018-025-06025-9)
Supplement: Supplementary file 3 — Supplementary Material 3 (453 KB) [file 18_2025_6025_MOESM3_ESM.docx]

**Supplementary material**

Contenido

[TABLES 1](#_Toc209185710)

[Table S1. 1](#_Toc209185711)

[Table S2. 2](#_Toc209185712)

[Table S3. 2](#_Toc209185713)

[Table S4 (in a separate excel file). 2](#_Toc209185714)

[Table S5. 3](#_Toc209185715)

[Table S6 (provided in a separate Excel file). 5](#_Toc209185716)

[FIGURES 6](#_Toc209185717)

[Supplemental Figure S1 7](#_Toc209185718)

[Supplemental Figure S2. 8](#_Toc209185719)

[REFERENCES 9](#_Toc209185720)

# TABLES

Table S1. Primers employed in the characterzation of the cellular models employed in this study

| **Name** | **Sequence (5´ to 3´)** | **Tm (° C)** | **PCR product size (bp)** |
| --- | --- | --- | --- |
| ***OCT3/4 Forward*** | GTTCTTCATTCACTAAGGAAGG | 58.4 | 101 |
| ***OCT3/4 Reverse*** | CAAGAGCATCATTGAACTTCAC | 58.4 |  |
| ***PAX6 Forward*** | GCTGCAAAGAAATAGAACATCC | 58.4 | 111 |
| ***PAX6 Reverse*** | TTGGCTGCTAGTCTTTCTCG | 58.4 |  |
| ***CRX Forward*** | CCCCAGTGTGGATCTGATG | 59.5 | 116 |
| ***CRX Reverse*** | CAAACAGTGCCTCCAGCTC | 59.5 |  |
| ***OPN1SW Forward*** | TTCTTCTCCAAGAGTGCTTGC | 59.5 | 97 |
| ***OPN1SW Reverse*** | CCTTCCCACACACCATCTTC | 60.5 |  |
| ***BEST1 Forward*** | TCAGTGTGGACACCTGTATGC | 61.2 | 84 |
| ***BEST1 Reverse*** | AAGCTGTACACCGCCACAG | 59.5 |  |
| ***RPE65 Forward*** | TTACTACGCTTGCACAGAGACC | 62.1 | 105 |
| ***RPE65 Reverse*** | GCCCCATTGACAGAGACATAG | 61.2 |  |
| ***MERTK Forward*** | TTGCAGCATTCAGGTCAAGGAAGC | 65.2 | 106 |
| ***MERTK Reverse*** | GGCTTGCAGCTGCTTGATTTGGTA | 65.2 |  |
| ***DCT Reverse*** | CTTTGCCGGCTATAATTGTG | 56.4 | 101 |
| ***DCT Forward*** | TCAAGGAATGGATGTTCTGC | 56.4 |  |
| ***ABCA4 Reverse*** | CATCCTGTTCCACCACCTCA | 60.5 | 113 |
| ***ABCA4 Forward*** | CTGTGTCCTCCAACATGGCT | 60.5 |  |
| ***MYO7A Forward*** | CTCAAACAGCCACTGCTCTACC | 64.1 | 122 |
| ***MYO7A Reverse*** | CTCATGGCTGTGTGGTACTTGG | 62.8 |  |
| ***GUSB Forward*** | AGAGTGGTGCTGAGGATTGG | 60.5 | 80 |
| ***GUSB Reverse*** | CCCTCATGCTCTAGCGTGTC | 62.5 |  |

Table S2. Details of antibodies employed in this study.

| **Target protein** | **Antibody** | **Application & dilution** |
| --- | --- | --- |
| β-Tubulin | Polyclonal rabbit anti-β-Tubulin (Abcam ab15568) | WB 1:1000 |
| Ezrin | Monoclonal mouse anti-Ezrin (Sigma Aldrich E8897) | ICC 1:250 |
| MYO7A | Monoclonal mouse anti-MYO7A (C-5) (Santa Cruz Biotechnology sc-74516) | WB 1:500 |
| MYO7A | Monoclonal rabbit anti-MYO7A (Abcam ab150386, EPR7497) | ICC 1:100 |
| ZO-1 | Polyclonal rabbit anti-ZO-1 (Invitrogen 40-2300) | ICC 1:500 |
| Nucleus | DAPI (Southern Biotechnologies 0100-20) | ICC 1:300  (of a 1:100 dilution) |
| Secondary | Goat anti-mouse Alexa Fluor 568 (Thermo Fisher Scientific A11031) | ICC 1:500 |
| Secondary | Goat anti-rat Alexa Fluor 488 (Thermo Fisher Scientific A11006) | ICC 1:500 |
| Secondary | Goat anti-mouse Alexa Fluor 488 (Thermo Fisher Scientific A11029) | WB 1:10.000 |
| Secondary | Goat anti-rabbit Alexa Fluor 568 (Thermo Fisher Scientific A11011) | ICC 1:500 |
| Secondary | Goat anti-rabbit IRDye 800 (Li-Cor LI 926-32211) | WB 1:10,000 |
| Secondary | Goat anti-rabbit Alexa Fluor 680 (Molecular Probes A-21076) | WB 1:10,000 |

Table S3. Summary of the microRNA associated to retina used to evaluate the retina specificity of tear derived EVs.

| **miRNA** | **Retina‑related evidence** | **Exclusively retina-expressed?** | **References** |
| --- | --- | --- | --- |
| **miR‑204‑5p** / **miR‑211** | Retinal development, optic cup, RPE/photoreceptor function, disease mutations | No | Conte, I., et al, 2010 (1); Wang, et al, 2010 (2); Barbato et al., 2017 (3); Bereimpour et al., 2021 (4); Du, et al., 2024 (5) |
| **miR‑181a/b** | High expression; involved in degeneration pathways | No | Ryan, et al., 2006 (6); Carrella, et al., 2015 (7), Carella et al, 2022 (8); Jiang et al., 2024 (9); Lopes da Acosta 2024 (10) |
| **miR‑125b** | Retinal cell lineage regulation; glaucoma/enriched in RPE | No | Zuzic et al., 2011 (11) |
| **miR‑21, miR‑100, miR‑146a, miR‑150** | Highly expressed in retina, roles in angiogenesis/inflammation | No | Liu et al., 2020 (12) |

Table S4 (in a separate excel file). Small non-coding RNA sequencing (sncRNA-seq) read counts across samples. The table includes average values for both control and patient groups, separated by sample type: culture media (EVs derived from iPSC-RPE) and tears (EVs from tear fluid). Relative expression levels are visualized using a color gradient, ranging from low (red) to high (green) expression. The lower section compiles miRNAs detected in all samples. miRNAs clearly associated with the retina are highlighted in **green**, while those with a less specific or indirect association are marked in **orange**.

Table S5. KEGG pathway enrichment analysis of differentially expressed miRNAs identified in EVs from tear fluid and hiPSC-derived RPE models when comparing patients and controls. The table lists the top 18 enriched pathways with p < 0.05. For each pathway, the number of genes involved (#genes) and the number of detected miRNAs predicted to regulate those genes (#miRNAs) are provided. Pathways shown in bold highlight those considered especially relevant to retinal biology and disease, with their potential roles in retinal structure, function, or pathology summarized in the final column.

| **KEGG pathway** | **p-value** | **#genes** | **#miRNAs** | **Relevance to Retina** |
| --- | --- | --- | --- | --- |
| **Fatty acid biosynthesis** | 0,00000 | 6 | 6 | Metabolism / Lipids. Photoreceptor outer segment renewal, RPE metabolism |
| **Hippo signaling pathway** | 0,00000 | 72 | 17 | Development & Differentiation. Retinal development, RPE differentiation, photoreceptor maintenance |
| Prion diseases | 0,00000 | 10 | 9 |  |
| **TGF-beta signaling pathway** | 0,00000 | 41 | 15 | Relevant pathway. Retinal fibrosis, RPE epithelial-mesenchymal transition |
| Proteoglycans in cancer | 0,00000 | 90 | 17 |  |
| **Adherens junction** | 0,00001 | 41 | 16 | Structural/Adhesion. RPE tight junctions, outer blood-retina barrier integrity |
| Pathways in cancer | 0,00002 | 172 | 17 |  |
| Thyroid hormone signaling pathway | 0,00003 | 59 | 17 |  |
| **mTOR signaling pathway** | 0,00003 | 37 | 14 | Signaling & Survival. Cell survival, apoptosis regulation, oxidative stress response in RPE and photoreceptors |
| **Signaling pathways regulating pluripotency of stem cells** | 0,00005 | 66 | 17 | Development & Differentiation iPSC-derived RPE differentiation, retinal regeneration models. |
| **Focal adhesion** | 0,00006 | 97 | 17 | Structural / Adhesion. RPE attachment to Bruch’s membrane, cytoskeleton dynamics. |
| ErbB signaling pathway | 0,00006 | 48 | 16 |  |
| Renal cell carcinoma | 0,00006 | 38 | 16 |  |
| **MAPK signaling pathway** | 0,00006 | 114 | 17 | Signaling & Survival. Stress response, inflammation, cell survival |
| **Regulation of actin cytoskeleton** | 0,00006 | 97 | 17 | Structural / Adhesion. RPE shape, phagocytosis, outer segment engulfment |
| Oxytocin signaling pathway | 0,00006 | 74 | 17 |  |
| **GABAergic synapse** | 0,00008 | 40 | 17 | Neural function / Synapse. Inhibitory signaling in retinal circuits |
| **Axon guidance** | 0,00011 | 57 | 17 | Neural function / Synapse. Retinal ganglion cell axon targeting during development |
| Long-term depression | 0,00012 | 31 | 15 |  |
|  |  |  |  |  |
| **Continuation table S5** |  |  |  |  |
| **KEGG pathway** | **p-value** | **#genes** | **#miRNAs** | **Relevance to Retina** |
| **ECM-receptor interaction** | 0,00033 | 32 | 15 | Structural / Adhesion. RPE-ECM interactions, retinal lamination |
| **Rap1 signaling pathway** | 0,00047 | 93 | 17 | Relevant Pathway. Cell adhesion, barrier function in RPE |
| Glioma | 0,00048 | 31 | 15 |  |
| **Wnt signaling pathway** | 0,00048 | 62 | 17 | Development & Differentiation. Retinal development, RPE differentiation, photoreceptor maintenance |
| **FoxO signaling pathway** | 0,00055 | 63 | 16 | Signaling & Survival. Oxidative stress and apoptosis regulation |
| Prostate cancer | 0,00069 | 44 | 14 |  |
| Colorectal cancer | 0,00074 | 32 | 15 |  |
| Estrogen signaling pathway | 0,00074 | 43 | 16 |  |
| Prolactin signaling pathway | 0,00105 | 34 | 15 |  |
| **Sphingolipid signaling pathway** | 0,00199 | 54 | 14 | Metabolism / Lipids. Apoptosis, inflammation, photoreceptor homeostasis |
| **p53 signaling pathway** | 0,00236 | 35 | 14 | Signaling & Survival. Apoptosis control; relevant in retinal cell death |
| Choline metabolism in cancer | 0,00236 | 49 | 15 |  |
| Adrenergic signaling in cardiomyocytes | 0,00239 | 59 | 16 |  |
| **PI3K-Akt signaling pathway** | 0,00246 | 135 | 17 | Signaling & Survival Cell survival, apoptosis regulation, oxidative stress response in RPE and photoreceptors. |
| Non-small cell lung cancer | 0,00297 | 27 | 14 |  |
| **Glutamatergic synapse** | 0,00559 | 50 | 17 | Neural function / Synapse. Photoreceptor to bipolar cell signaling |
| Bacterial invasion of epithelial cells | 0,00597 | 37 | 16 |  |
| **Endocytosis** | 0,00678 | 87 | 17 | Relevant Pathway. Photoreceptor outer segment phagocytosis, protein quality control |
| Ras signaling pathway | 0,00723 | 89 | 17 |  |
| Insulin signaling pathway | 0,00776 | 62 | 16 |  |
| Chronic myeloid leukemia | 0,00791 | 36 | 15 |  |
| **Neurotrophin signaling pathway** | 0,00792 | 56 | 17 | Development & Differentiation. Retinal neuron survival, synaptic plasticity |
| **Gap junction** | 0,00835 | 44 | 17 | Structural / Adhesion. Retinal cell communication, homeostasis |
| cGMP-PKG signaling pathway | 0,00921 | 69 | 17 |  |
| Melanoma | 0,00993 | 34 | 15 |  |
| **Continuation table S5** |  |  |  |  |
| **KEGG pathway** | **p-value** | **#genes** | **#miRNAs** | **Relevance to Retina** |
| **AMPK signaling pathway** | 0,01364 | 56 | 15 | Metabolism / Lipids. Energy sensing, stress response in retinal cells |
| mRNA surveillance pathway | 0,02103 | 41 | 16 |  |
| Long-term potentiation | 0,02103 | 32 | 17 |  |
| **Ubiquitin mediated proteolysis** | 0,02414 | 59 | 17 | Relevant Pathway. Photoreceptor outer segment phagocytosis, protein quality control |
| Small cell lung cancer | 0,03168 | 38 | 14 |  |
| Platelet activation | 0,03181 | 52 | 17 |  |
| Endometrial cancer | 0,03291 | 26 | 15 |  |
| cAMP signaling pathway | 0,03743 | 79 | 17 |  |
| Gastric acid secretion | 0,04299 | 34 | 16 |  |

Table S6 (provided in a separate Excel file). **Comparison of our data with those published by Getachew *et al*, 2025.** Comparison of normalized EV-sncRNA expression levels between the iPSC-RPE model and EVs isolated from tears. The first two columns show data from our study: **patients** (left column) and **controls** (middle column). The third column shows data reported by Getachew et al. (2025) (13). miRNAs that were **downregulated** in the RPE models used by Getachew et al. are highlighted in **red**, while **upregulated** miRNAs are highlighted in **green**. Matches between our dataset and the findings of Getachew et al. are marked using the same color coding in our data columns. Additionally, sncRNAs detected in **both** our patient and control samples are highlighted in **yellow**. A heatmap summarizing the overall data from our study is included below and also appears as **Table 3b**.

# FIGURES


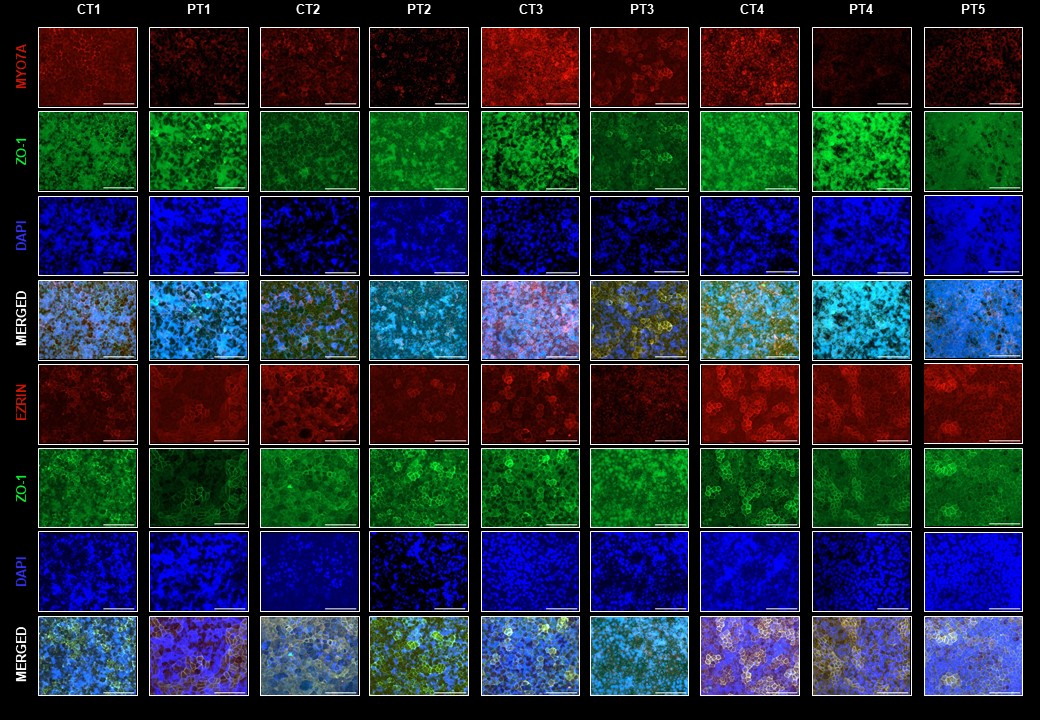


Supplemental Figure S1**. Immunocytochemistry of RPE monolayers derived from control (CT) and USH1B patient (PT) iPSC lines. Representative images show the expression and localization of the RPE markers MYO7A (red, top panel) and Ezrin (red, second panel from bottom), along with the tight-junction protein ZO-1 (green) and nuclear counterstain DAPI (blue). Each column corresponds to an individual control (CT1–CT4) or patient-derived line (PT1–PT5). The fourth row in each staining set displays the merged channels (MYO7A or Ezrin + ZO-1 + DAPI). ZO-1 delineates tight junctions, while MYO7A and Ezrin highlight the localization of RPE-specific proteins. Although the representative images show variable background intensity, likely due to imaging through the transwell membrane, the overall distribution of Ezrin and ZO-1 overlaps within each sample, and no consistent differences were observed between controls and patient-derived cells. MYO7A signal intensity was reduced in patient cells carrying pathogenic variants. Scale bar: 100 µm.**

**
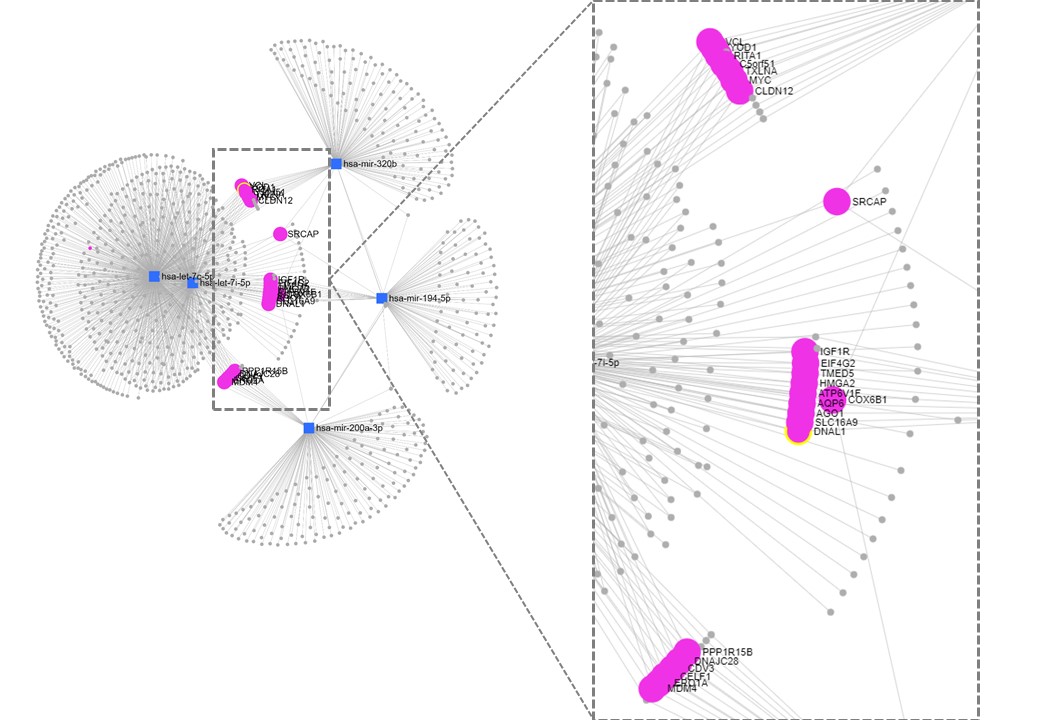
**

Supplemental Figure S2. Input process for **miRNet** analysis, incorporating the miRNAs found to be dysregulated in the analysis shown in **Figure 4c, Figure 4d**, and **Supplementary Table S3**. miRNet predicted several shared targets for the following miRNAs: **hsa-let-7c-5p**, **hsa-let-7i-5p**, **hsa-miR-320b**, **hsa-miR-194-5p**, and **hsa-miR-200a-3p**. These shared targets are displayed on the right side of the figure, with a **zoomed-in view** to enhance readability. Targets highlighted in **pink** were selected for further analysis, as they were predicted to be **common targets shared by all the miRNAs** included in the analysis.

*Source:* https://www.mirnet.ca/upload/MirUploadView.xhtml

# REFERENCES

1. Conte I, Carrella S, Avellino R, Karali M, Marco-Ferreres R, Bovolenta P, et al. miR-204 is required for lens and retinal development via Meis2 targeting. Proc Natl Acad Sci U S A. 31 de agosto de 2010;107(35):15491-6.

2. Wang FE, Zhang C, Maminishkis A, Dong L, Zhi C, Li R, et al. MicroRNA-204/211 alters epithelial physiology. FASEB J. mayo de 2010;24(5):1552-71.

3. Barbato S, Marrocco E, Intartaglia D, Pizzo M, Asteriti S, Naso F, et al. MiR-211 is essential for adult cone photoreceptor maintenance and visual function. Sci Rep. 5 de diciembre de 2017;7(1):17004.

4. Bereimipour A, Najafi H, Mirsane ES, Moradi S, Satarian L. Roles of miR-204 in retinal development and maintenance. Experimental Cell Research. 1 de septiembre de 2021;406(1):112737.

5. Du SW, Komirisetty R, Lewandowski D, Choi EH, Panas D, Suh S, et al. Conditional deletion of miR-204 and miR-211 in murine retinal pigment epithelium results in retinal degeneration. J Biol Chem. junio de 2024;300(6):107344.

6. Ryan DG, Oliveira-Fernandes M, Lavker RM. MicroRNAs of the mammalian eye display distinct and overlapping tissue specificity. Mol Vis. 17 de octubre de 2006;12:1175-84.

7. Carrella S, D’Agostino Y, Barbato S, Huber‐Reggi SP, Salierno FG, Manfredi A, et al. miR‐181a/b control the assembly of visual circuitry by regulating retinal axon specification and growth. Developmental Neurobiology. 11 de junio de 2015;75(11):1252.

8. Carrella S, Di Guida M, Brillante S, Piccolo D, Ciampi L, Guadagnino I, et al. miR-181a/b downregulation: a mutation-independent therapeutic approach for inherited retinal diseases. EMBO Mol Med. 8 de noviembre de 2022;14(11):e15941.

9. Jiang B, Hong N, Zhang L, Xu B, He Q, Qian X, et al. MiR-181a-5p may regulate cell proliferation and autophagy in myopia and the associated retinopathy. Exp Eye Res. abril de 2024;241:109829.

10. Costa BL da, Quinn PMJ, Wu WH, Liu S, Nolan ND, Demirkol A, et al. Targeting miR-181a/b in retinitis pigmentosa: implications for disease progression and therapy. Cell Biosci. 21 de mayo de 2024;14(1):64.

11. Zuzic M, Arias JER, Wohl SG, Busskamp V. Retinal miRNA Functions in Health and Disease. Genes. 17 de mayo de 2019;10(5):377.

12. Liu CH, Huang S, Britton WR, Chen J. MicroRNAs in Vascular Eye Diseases. Int J Mol Sci. 19 de enero de 2020;21(2):649.

13. Getachew H, Mehrotra S, Kaur T, Fernandez-Godino R, Pierce EA, Garita-Hernandez M. The RNA content of extracellular vesicles from gene-edited PRPF31 +/- hiPSC-RPE show potential as biomarkers of retinal degeneration. Mol Ther Methods Clin Dev. 12 de junio de 2025;33(2):101452.
